# LncRNA profile study reveals four-lncRNA signature associated with the prognosis of patients with anaplastic gliomas

**DOI:** 10.18632/oncotarget.12624

**Published:** 2016-10-13

**Authors:** Wen Wang, Fan Yang, Lu Zhang, Jing Chen, Zheng Zhao, Haoyuan Wang, Fan Wu, Tingyu Liang, Xiaoyan Yan, Jiye Li, Qing Lan, Jiangfei Wang, Jizong Zhao

**Affiliations:** ^1^ Department of Neurosurgery, Beijing Tiantan Hospital, Capital Medical University, Beijing, China; ^2^ Department of Neurosurgery, The Second Affiliated Hospital of Soochow University, Suzhou, China; ^3^ Department of Molecular Neuropathology, Beijing Neurosurgical Institute, Capital Medical University, Beijing, China; ^4^ Beijing Neurosurgical Institute, Capital Medical University, Beijing, China; ^5^ Department of Ophthalmology, School of Medicine, Shandong University, Jinan, China; ^6^ Department of Neurosurgery, Zhujiang Hospital, Southern Medical University, Guangzhou, China; ^7^ Chinese Glioma Cooperative Group (CGCG), Beijing, China; ^8^ China National Clinical Research Center for Neurological Diseases, Beijing, China

**Keywords:** signature, prognosis, anaplastic glioma, lncRNA, RNA microarray

## Abstract

Anaplastic glioma is Grade III and the median overall survival is about 37.6 months. However, there are still other factors that affect the prognosis for anaplastic glioma patients due to variable overall survival. So we screened four-lncRNA signature (AGAP2-AS1, TPT1-AS1, LINC01198 and MIR155HG) from the lncRNA expression profile from the GSE16011, CGGA and REMBRANDT datasets. The patients in low risk group had longer overall survival than high risk group (median OS 2208.25 vs. 591.30 days; P < 0.0001). Moreover, patients in the low risk group showed similar overall survival to Grade II patients (P = 0.1669), while the high risk group showed significant different to Grade IV (P = 0.0005) with similar trend. So based on the four-lncRNA, the anaplastic gliomas could be divided into grade II-like and grade IV-like groups. On the multivariate analysis, it showed the signature was an independent prognostic factor (P = 0.000). The expression of four lncRNAs in different grades showed that AGAP2-AS1, LINC01198 and MIR155HG were increased with tumor grade, while TPT1-AS1 was decreased. Knockdown of AGAP2-AS1 can inhibit the cell proliferation, migration and invasion, while increase the apoptosis cell rates in vitro. In conclusion, our results showed that the four-lncRNA signature has prognostic value for anaplastic glioma. Moreover, clinicians should conduct corresponding therapies to achieve best treatment with less side effects for two groups patients.

## INTRODUCTION

Glioma is the most common brain tumor and it has high morality and recurrence rate [1]. According to the World Health Organization (WHO) classification, glioma is classified into four grades, as which the anaplastic glioma (AG) is Grade III [2], including anaplastic astrocytoma (AA), anaplastic oligodendroglioma (AO) and anaplastic oligoastrocytoma (AOA). AGs comprise 6–10% of all primary brain tumors [3] and the median overall survival (OS) is about 37.6 months [4]. Current evidence suggests that the progression of gliomas may involve the accumulation of multiple genetic alterations, such as isocitrate dehydrogenase (IDH) mutation, codeletion of chromosomal arms 1p and 19q, methylation of O(6)-methylguanine-DNA methyl transferase (MGMT) promoter and alpha thalassemia/mental retardation syndrome X-linked (ATRX) mutations or loss, et al [5]. However, due to variable OS of patients with AGs, there are still other factors that affect the prognosis for AG patients.

Long noncoding RNA (LncRNA) is defined as longer than 200nucleotides without protein-coding ability [6]. Many studies have revealed a wide range of functional activities of lncRNAs [7, 8], including chromatin remodeling, transcriptional control and post-transcriptional processing, et al. The dysregulation of lncRNAs might contribute towards glioma pathogenesis, such as cellular proliferation and apoptosis [9–12]. Aberrant expressions of lncRNAs may have prognostic value for AG patients and can be exploited as potential therapeutic targets [13, 14].

In our study, we obtained GSE16011 dataset as training set while the Chinese Glioma Genome Atlas (CGGA) and the Repository for Molecular Brain Neoplasia Data (REMBRANDT) datasets as validated sets. A total of 183 (GSE 80; CGGA 36; REMBRANDT 67) AG patients were included. Using Cox regression analysis and receiver operating characteristic (ROC) curve, we identified four-lncRNA signature (AGAP2-AS1, TPT1-AS1, LINC01198 and MIR155HG) which have prognostic value for AGs. Based on the median risk score of the signature, AGs could be divided into low risk and high risk groups. The patients in low risk group had longer OS than high risk group.

## RESULTS

### Identification and validation of four-lncRNA signature from the three datasets

The lncRNAs list and their expression profiles were extracted from each microarray dataset by using lncRNA expression profile mining [15]. A total of 572 lncRNAs were identified from the three datasets. Then, we collected 80 anaplastic glioma patients from GSE16011 dataset as training set. 45 probes (33 lncRNAs) were pinpointed on univariate Cox analysis and the top 10 prognostic probes were listed in Table 1 ranked ascendingly by their p value. By applying time-dependent ROC curve, we could get a series of area under the curve (AUC) (0.909, 0.907, 0.899, 0.942, 0.913, 0.917, 0.918) by adding genes in the list from top to bottom to the signature [16]. The maximal AUC (0.942) was achieved from the top 4 probes (4 lncRNAs) (Figure 3A). The four lncRNAs were AGAP2-AS1, TPT1-AS1, LINC01198 and MIR155HG.

**Table 1:** Top ten prognostic probes identified from Cox regression analysis from GSE16011 dataset

Gene Symbol	Probe	HR	low95	high95	P-value
AGAP2-AS1	1555907_at	1.712	1.410	2.079	5.70E-08
TPT1-AS1	227709_at	0.353	0.237	0.525	2.75E-07
LINC01198	1553614_a_at	1.630	1.334	1.990	1.68E-06
MIR155HG	229437_at	1.725	1.367	2.176	4.31E-06
TPT1-AS1	227710_s_at	0.278	0.158	0.491	1.03E-05
LINC00476	1557788_a_at	0.505	0.372	0.687	1.29E-05
LINC00944	1560573_at	3.574	2.007	6.365	1.52E-05
LOC100130691	231540_at	0.102	0.036	0.291	2.02E-05
LINC00152	1552258_at	19.315	4.916	75.881	2.22E-05
WAC-AS1	233013_x_at	0.274	0.149	0.504	3.14E-05

**Figure 1:**
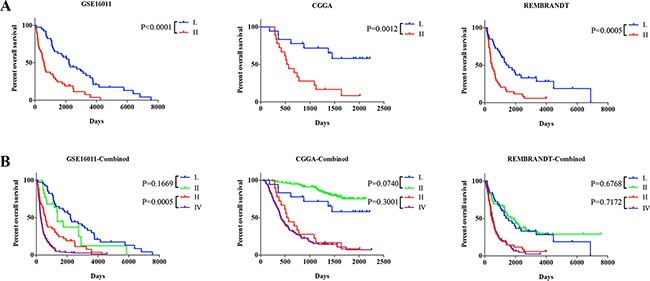
Comparison of prognostic value among different groups or Grades **A.** Overall survival among AG patients in different groups stratified by low and high risk group in three datasets. **B.** Overall survival among AG patients in different groups stratified by the signature and Grades (Grade II and Grade IV) in three datasets. *P < 0.05, **P < 0.01, ***P < 0.001.

Then, we developed a four-lncRNA signature using a risk score method [17–19]. We divided two groups (low risk and high risk groups) based on the median risk score (0.2067). The patients in low risk group had longer OS than high risk group (median OS 2208.25 vs. 591.30 days; P < 0.0001; Figure 1A). We further validated the four-lncRNA signature in two additional datasets using the same β value. It showed similar results (median OS 1432 vs. 548.5 days; P = 0.0012) (median OS 1276 vs. 465.5 days; P = 0.0005; Figure 1A) between two groups, respectively.

### Low risk and high risk groups showed similar overall survival to Grade II and IV gliomas respectively

We divided the AGs into two groups based on the median risk score (0.2067). Furthermore, we assessed the prognostic value of this signature compared with Grade II and IV gliomas.

22 Grade II glioma patients and 142 Grade IV glioma patients from GSE16011 were included in our analysis. The patients in the low risk group showed similar OS to Grade II patients (P = 0.1669), while the high risk group showed significant different to Grade IV (P = 0.0005) with similar trend (Figure 1B). We further validated the findings in CGGA and REMBRANDT datasets (Figure 1B). The low risk group showed similar OS to Grade II (P = 0.0740; P = 0.6768) and the high risk group also showed similar OS to Grade IV (P = 0.3001; P = 0.7172), respectively. It indicated that OS of two groups were similar to Grade II and IV glioma patients'. So based on the four-lncRNA, we could divide grade II-like and grade IV-like AGs.

### Clinical and molecular features of low and high risk AG patients

**Figure 2:**
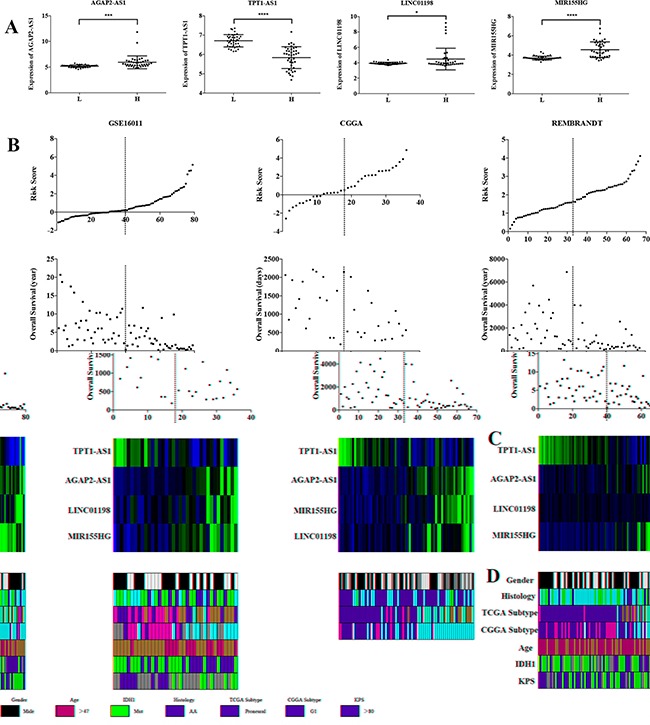
Distribution of risk score, OS, gene expression and clinical or molecular pathological features in three datasets **A.** The expression of four lncRNAs in low risk and high risk groups in scatter dot plot. **B.** Distribution of risk score and OS. The black dotted lines in the middle of each graph represent the gene signature cutoff (median risk score). **C.** Heat map of the expression of four lncRNAs in low risk and high risk groups. Rows represent corresponding genes, and columns indicate corresponding patients. **D.** Clinical or molecular pathological features in three datasets. Rows represent corresponding items (gender, histology, TCGA subtype, CGGA subtype, age, IDH1 and KPS).

The expression levels of the four lncRNAs showed significant difference between low risk and high risk groups (Figure 2A, C). TPT1-AS1 was higher expressed in low risk group, so we considered it as a protective lncRNA. The other three lncRNAs (AGAP2-AS1, LINC01198 and MIR155HG) were higher expressed in high risk group, so we considered them as risky genes. We observed that AG patients in the high risk group had shorter OS than low risk group (Figure 2B). The related clinical information such as gender, histology, TCGA subtype, CGGA subtype, age, IDH1 mutation and KPS were obtained from GSE16011 database. Patients in high risk group tended to display older age (>47 years), classical and mesenchymal TCGA subtype, G3 CGGA subtype and lower KPS (Figure 2D). Moreover, we further validated in CGGA and REMBRANDT databases (Figure 2D).

We assessed the independence of the four-lncRNA signature in the GSE16011 dataset. In the univariate cox regression analysis, the signature was significant associated with the OS (P = 0.000) along with age and KPS status. Moreover, it showed the signature was an independent prognosis factor (P = 0.000) on the multivariate analysis (Table 2). In two additional datasets, the results indicated the similar results that the risk score was an independent prognostic factor (P = 0.030; P = 0) (Supplementary Table S1).

**Table 2:** Clinicopathologic factors associated with OS in the Cox regression analysis for patients from the GSE16011 microarray dataset

Variable	Univariate Cox		Multivariate Cox	
	p-value	HR	p-value	HR
Age	0.000	1.035	0.041	1.025
Gender	0.957	1.014		
KPS	0.012	0.986	0.347	0.994
IDH1 Mutation	0.288	0.744		
Chemotherapy	0.939	0.976		
Risk Score	0.000	1.858	0.000	1.923

Gender, male 1, female 2; IDH1 mutation status, mutated 1, wild-type 0; Chemotherapy, Yes 1, No 0.

**Figure 3:**
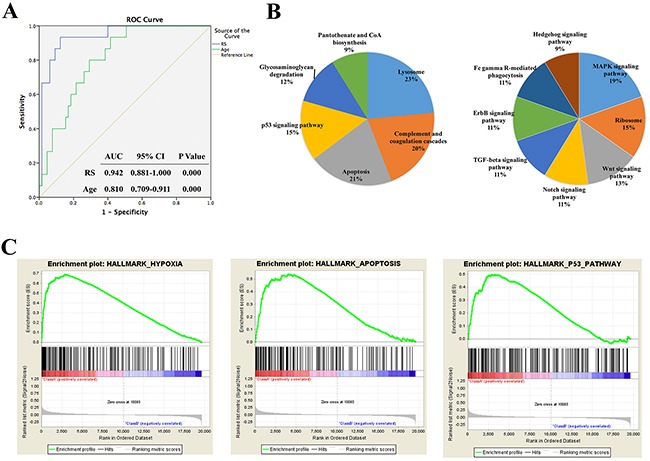
ROC curve of the four-lncRNA signature and functional annotation of each risk group **A.** ROC curve of the four-lncRNA signature. **B.** KEGG pathway analysis of the different genes in two groups. Circle area: the percent of gene counts. **C.** Three representative plots of GSEA.

Furthermore, the four-lncRNA signature (AGAP2-AS1, TPT1-AS1, LINC01198 and MIR155HG) achieved the highest accuracy for predicting AG patients by time-dependent ROC curve analysis (AUC = 0.942), following the age (AUC = 0.810) (Figure 3A). It indicated that the signature can specially predict the prognosis of AGs independently.

### Function exploration of the signature for prognosis of AGs

**Figure 4:**
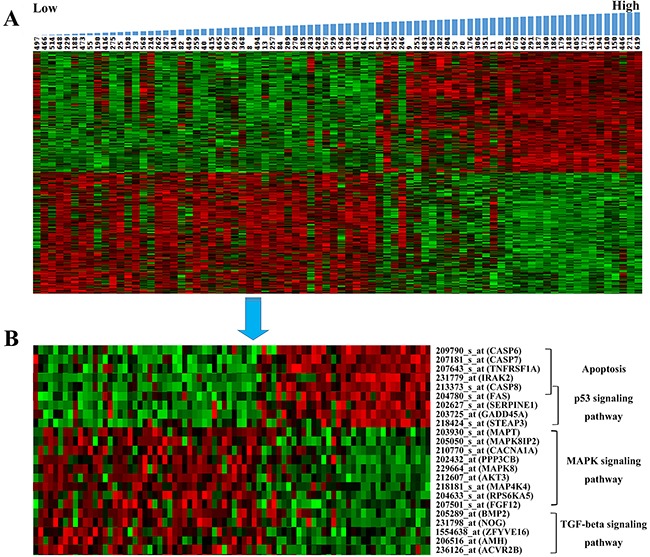
Hierarchical clustering analysis of mRNA expression profiles **A.** Hierarchical clustering analysis of mRNA expression profiles based on the top 1000 genes. **B.** Hierarchical clustering analysis of important genes related to four pathways.

To explain the different prognosis of AGs divided by the signature, we performed SAM analysis between two groups using R software (False Discovery Rate, FDR < 0.01). The top 500 probes were selected from positive and negative group, respectively. Then the expression of 1000 probes were showed in Figure 4A using a hierarchical clustering analysis.

Moreover, we performed Kyoto Encyclopedia of Genes and Genomes (KEGG) pathways analysis using DAVID (The Database for Annotation, Visualization and Integrated Discovery). The results showed that selected up-regulated genes were enriched in lysosome, complement and coagulation cascades, apoptosis and p53 signaling pathway, et, al. While the down-regulated genes were enriched in MAPK signaling pathway, ribosome, Wnt signaling pathway, Notch signaling pathway and TGF-beta signaling pathway, et, al (Figure 3B). We further performed Gene Set Enrichment Analysis (GSEA) for functional annotation [20, 21], the results showed that the differential expressed genes were enriched in hypoxia, apoptosis and p53 pathway (Figure 3C). Moreover, the important genes related to apoptosis, p53signaling pathway, MAPK signaling pathway and TGF-beta signaling pathway were indicated in Figure 4B.

Finally, we explored the interaction properties with proteins of the four lncRNAs using CLIPdb (http://clipdb.ncrnalab.org), which was developed to predict lncRNA-binding proteins using the CLIP-seq data [22]. We entered the lncRNAs into the Binding Target Search form and identified lncRNA-binding proteins. However, we failed to predict the protein which is interacted with LINC01198. The results showed in Table 3. We further validated these findings using starBase V2.0 [23, 24]. The results were partly similar to CLIPdb', so the lncRNA-binding proteins we predicted in Table 3 may just provide clues for further study of the four lncRNAs. It partially explained the poor OS of patients in high risk group.

**Table 3:** Predictive proteins interacted with the identified lncRNAs

Protein	Identified lncRNAs	Function of interactive protein
AGO2	TPT1-AS1	Cell invasion, proliferation, apoptosis, and cell cycle
	MIR155HG	
CPSF7	TPT1-AS1	Cell proliferation
	MIR155HG	
ELAVL1	AGAP2-AS1	Cell proliferation and invasion
	TPT1-AS1	
	MIR155HG	
FUS	AGAP2-AS1	Cell proliferation, apoptosis and Cell cycle
	TPT1-AS1	
	MIR155HG	

The lncRNA-binding proteins were predicted by CLIPdb.

### The expression of the four-lncRNA signature in different grades

**Figure 5:**
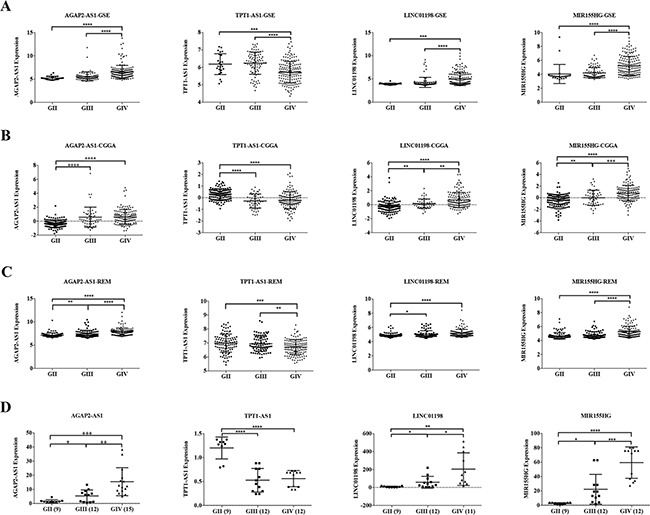
The expression of four-lncRNA signature in different grades and qRT-PCR validation **A.** GSE16011 dataset. **B.** CGGA dataset. **C.** REMBRANDT dataset. **D.** qRT-PCR validation. *P < 0.05, **P < 0.01, ***P < 0.001, **** P < 0.0001.

We assessed the expression of four lncRNAs in different grades and the results showed that AGAP2-AS1, LINC01198 and MIR155HG were increased with tumor grade, while TPT1-AS1 was decreased in GSE16011 dataset (Figure 5A). The results were similar in two additional datasets (CGGA, REMBRANDT) (Figure 5B, C). We further validated these findings using quantitative real time polymerase chain reaction (qRT-PCR) in an independent group (Grade II 9, Grade III 12, Grade IV 15) (Figure 5D). The primers were listed in Supplementary Table S2.

### Knockdown AGAP2-AS1 suppresses cell proliferation, migration and invasion, while increases apoptosis cell rates *in vitro*

**Figure 6:**
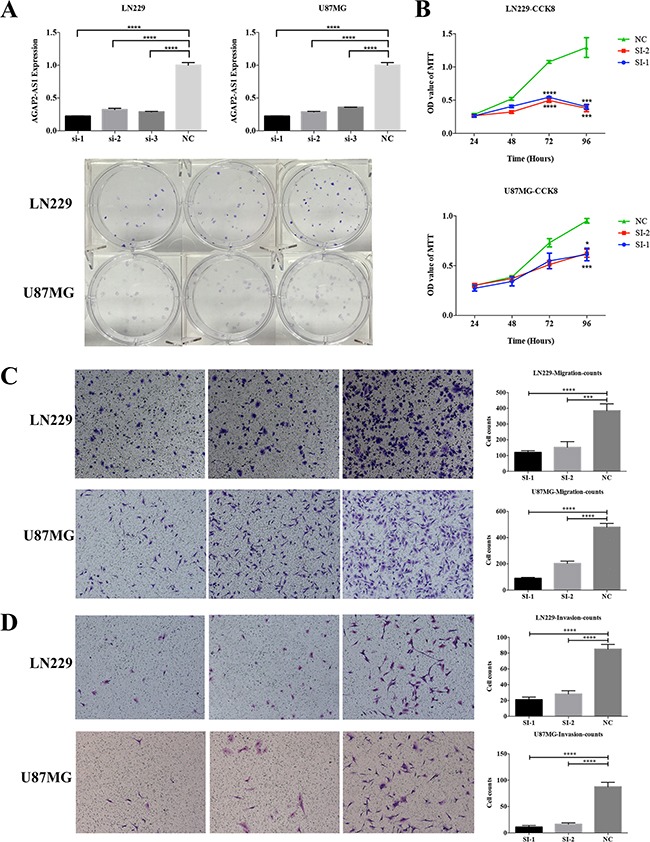
AGAP2-AS1 knockdown inhibits cell proliferation, migration and invasion *in vitro* **A.** The knockdown result of AGAP2-AS1 by siRNAs in LN229 and U87MG cell lines. **B.** The cell proliferation was inhibited by AGAP2-AS1 knockdown. **C.** The cell migration was inhibited by AGAP2-AS1 knockdown. **D.** The cell invasion was inhibited by AGAP2-AS1 knockdown. *P < 0.05, **P < 0.01, ***P < 0.001, **** P < 0.0001.

To determine the functional role of AGAP2-AS1 in glioma, we assessed the effects of knockdown of AGAP2-AS1 with siRNAs on cell proliferation, migration and invasion. Three AGAP2-AS1 specific siRNAs were listed in Supplementary Table S2. We evaluated their knockdown efficiency in LN229 and U87MG (Figure 6A), si-1 and si-2 were found to have a higher silencing efficiency.

**Figure 7:**
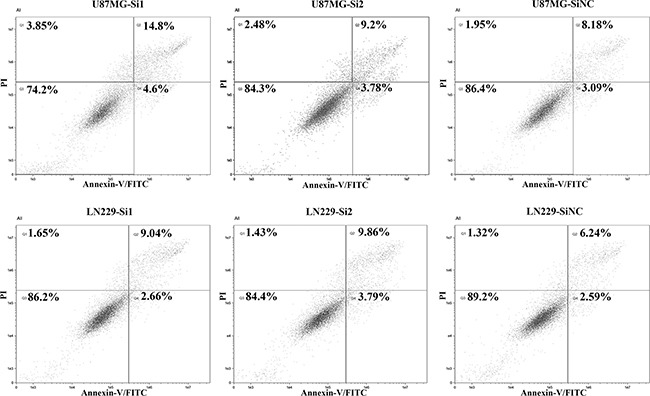
Histograms of flow cytometry analysis for apoptosis (Q2: Late apoptosis; Q4: Early apoptosis).

The results showed that knockdown of AGAP2-AS1 can inhibit the cell proliferation in LN229 and U87MG using CCK8 assay (Figure 6B). Moreover, down-regulation of AGAP2-AS1 can also suppress cell migration and invasion (Figure 6C, D). Annexin-V staining showed that apoptosis cell rates were increased with AGAP2-AS1 knockdown in LN229 and U87MG (Figure 7).

## DISCUSSION

The classification of AGs based on mRNA expression profiling has been reported preciously [17]. However, with the functions of lncRNAs exploring, there are few studies focus on the classification of AGs based on lncRNAs expression profiling.

We mined lncRNAs data from three datasets and selected GSE16011 as a training dataset. After Cox regression analysis and time-dependent ROC curve, we screened four-lncRNA signature (AGAP2-AS1, TPT1-AS1, LINC01198 and MIR155HG) for the prognosis of AGs. Moreover, we validated the finding in CGGA and REMBRANDT datasets. We divided two groups based on the median risk score which is developed by a widely used approach [17–19]. We observed that AG patients in the high risk group had shorter OS than low risk group. Furthermore, we assessed the prognostic value of this signature compared with Grade II and IV gliomas. It indicated that the signature can specially divide grade II-like and grade IV-like AGs.

The WHO classification based on morphological criteria (nuclear atypia, mitoses, vascular proliferation and necrosis) [25, 26]. However, the four-lncRNA signature can specially divide the AGs into two groups. Clinicians should pay more attention to the treatment of AG patients to achieve best prognosis with less side effects.

Differential expressed genes of low risk and high risk groups were performed KEGG pathways analysis and the results showed that up-regulated genes were enriched in lysosome, complement and coagulation cascades, apoptosis and p53 signaling pathway, et, al. While the down-regulated genes were enriched in MAPK signaling pathway, ribosome, Wnt signaling pathway, Notch signaling pathway and TGF-beta signaling pathway, et, al (Figure 3B). Moreover, GSEA showed that the differential expressed genes were enriched in hypoxia, apoptosis and p53 pathway (Figure 3C). We further explored the interaction properties with proteins of the four lncRNAs using CLIPdb and starBase V2.0 (Table 3). AGO2 can play a role of RNA interference by encoding a member of argonaute family of proteins [27] and it predictively interacted with TPT1-AS1 and MIR155HG. It is reported that AGO2 can regulate tumor invasion, proliferation, apoptosis and cell cycle in glioma, cervical and prostate cancer [28–30]. CPSF7 can play a regulatory role in polyA site selection [31] and it is found to be significantly associated with tumor recurrence in breast cancer recently [32]. ELAVL1 (HuR) is an RNA-binding protein and the decreased of ELAVL1 can resist cell proliferation and invasion in ovarian and prostate cancer [33, 34]. FUS belongs to the FET family and it encodes a multifunctional RNA-binding protein which is highly associated with tumor progression. The overexpression of FUS can promote growth inhibition and apoptosis of prostate cancer [35, 36].

We also assessed the expression of four lncRNAs in different grades and the results showed that AGAP2-AS1, LINC01198 and MIR155HG were increased with tumor grade, while TPT1-AS1 was decreased (Figure 5). Moreover, knockdown of AGAP2-AS1 can inhibit the cell proliferation, migration and invasion, while increase the apoptosis cell rates in LN229 and U87MG (Figure 6, and 7).

There are limitations in our manuscript. Only a total of 183 primary AGs were enrolled, which was a small sample and only a part of lncRNAs included in our analysis from microarray data. Moreover, functions of the identified four lncRNAs were only predicted by DAVID, CLIPdb and starBase V2.0, so the RNA-binding proteins we predicted in Table 3 may just provide clues for further study of the four lncRNAs. However, identified lncRNAs may exert their functions through similar mechanisms to the presumed interacted proteins.

In conclusion, our results showed that the four-lncRNA signature has prognosis value for patients with AGs. Due to the different prognosis in two groups, clinicians should conduct corresponding therapies to achieve best treatment with less side effects. Moreover, knockdown of AGAP2-AS1 can inhibit the cell proliferation, migration and invasion, while increase the apoptosis cell rates in vitro.

## MATERIALS AND METHODS

### Patients and datasets

The training dataset GSE16011 included 244 patients (Grade II 22; Grade III 80; Grade IV 142) was downloaded from the Gene Expression Omnibus (GEO). 282 patients (Grade II 120; Grade III 36; Grade IV 126) from the Chinese Glioma Genome Atlas (CGGA) and 241 patients (Grade II 69; Grade III 67; Grade IV 105) from the Repository for Molecular Brain Neoplasia Data (REMBRANDT) were included in our analysis as validated datasets. Patients were eligible for the study if their diagnosis was established histologically by two neuropathologists according to the 2007 WHO classification guidelines. All patients' clinical information was download from each website.

### LncRNA profile mining

The lncRNA profile was achieved by the established mining method [15]. Genes were identified as protein-coding genes or noncoding genes based on their Refseq IDs or Ensembl IDs. GSE16011 was settled on Affymetrix HG-U133 Plus 2.0 platform, so we further filtered them by removing pseudogenes, rRNAs, tRNAs, snRNAs, snoRNAs, and other short non-coding RNAs and retained only the long noncoding genes in NetAffx Annotation files. We only retained the 572 lncRNAs that were represented in all three datasets (GSE16011, CGGA and REMBRANDT) to ensure the validity of the gene signatures.

### Signature development

The risk score was developed as previously reported [17–19, 37], based on a linear combination of the lncRNA expression level (expr) weighted by the regression coefficient (β) derived from the univariate Cox regression analysis. The risk score for each patient was calculated as follows:

Risk score = β_gene1_ × expr_gene1_ + β_gene2_ × expr_gene2_+ ··· + β_genen_ × expr_genen_

We divided anaplastic gliomas patients into low risk and high risk groups using the median risk score as cutoff point.

### Quantitative real time polymerase chain reaction

Quantitative real time polymerase chain reaction (qRT-PCR) was performed to detect the expression levels of AGAP2-AS1, TPT1-AS1, LINC01198 and MIR155HG. Total RNAs were extracted from frozen tissues or cell lines using TRIzol reagent (Invitrogen, CA, USA) and then reversely transcribed using ReventAid First Strand cDNA Synthesis Kit (Thermo, MA, USA) in accordance with the manufacturer's instructions. Glyceralde-hyde 3-phosphate dehydrogenase (GAPDH) was used as an internal control and all the primer sequences are shown in Supplementary Table S2. qRT-PCR was performed using SYBR Select Master Mix (Applied Biosystems, CA, USA).

### Cell proliferation, migration and invasion assays

Cell proliferation was assayed using Cell Counting Kit-8 (Dojindo, Japan) according to the manufacturer's instructions. Cells were seeded at a density of 2000 cells per well in 96 well plates. After AGAP2-AS1 siRNAs and negative control (NC) transfection for 24 h, cells were evaluated by measuring the absorbance at 450 nm. Cells were transfected with siRNAs targeting AGAP2-AS1 for 48 h and then about 1000 cells were plated in each well of the 12-well plate and maintained for 2 weeks to form colony.

Cell migration assay were performed by using Transwell insert chambers (8μm pore size, Corning, USA). About 2 × 10^4^ cells were seeded into the upper chamber in serum free medium in triplicate. The lower chamber was filled with 600μl medium containing 10% fetal bovine serum (FBS). After incubation for 4 h, cells migrating to the lower surface of membrane were fixed using paraformalclehyde and stained with 0.1% crystal violet. For invasion assay, Matrigel Invasion Chambers in the 24-well plates were used.

### Flow cytometry analysis for apoptosis

For apoptosis assay, cells were harvested after transfection with AGAP2-AS1 siRNAs for 36 h, and then processed to stain with Annexin V-FITC/Propidium Iodide kit (Beijing 4A Biotech Co., Ltd, China) according to the manufacturer's instructions. The flow cytometry was performed by ImageStreamX Mark II instrument and analyzed with IDEA software.

### Statistical analysis

We firstly excluded patients without survival data or ≤ 30 days because they may die of other reasons. Then we performed Cox analysis and the probes were ranked ascendingly by their p value. By applying time-dependent ROC curve, we could get a series of area under the curve (AUC) by adding genes in the list from top to bottom to the signature.

The significance analysis of microarray (SAM) and Cox regression analysis was calculated using R software (version 3.2.3) with the samr and survival packages. The univariate, multivariate cox regression analysis and ROC curve were performed by SPSS software (version 22; SPSS Inc., Chicago, IL, USA). The Kaplan-Meier curve was performed by GraphPad Prism 6 (GraphPad Software Inc., La Jolla, CA, USA). A two-sided P value of < 0.05 was regarded as statistically significant.

### LncRNA-binding protein interaction

The interaction proteins of the identified four lncRNAs were analyzed by using the public CLIPdb (http://clipdb.ncrnalab.org) and starBase v2.0 (http://starbase.sysu.edu.cn) [22–24]. CLIPdb described RBP-RNA interactions based on 395 publicly available CLIP-seq data sets and it can provide high-resolution RBP binding sites both in mRNA and non-coding RNA [22]. starBase v2.0 was designed for decoding RBP-RNA interactions from CLIP-seq experimentally and it can provide the number of lncRNA binding sites with certain RBPs [23, 24].

## SUPPLEMENTARY MATERIALS TABLES





## References

[1] Nikiforova MN, Hamilton RL (2011). Molecular diagnostics of gliomas. Arch Pathol Lab Med.

[2] Louis DN, Ohgaki H, Wiestler OD, Cavenee WK, Burger PC, Jouvet A, Scheithauer BW, Kleihues P (2007). The 2007 WHO classification of tumours of the central nervous system. Acta Neuropathol.

[3] Simonetti G, Gaviani P, Innocenti A, Botturi A, Lamperti E, Silvani A (2014). Update on treatment strategies for anaplastic glioma: a review of literature. Neurol Sci.

[4] Yang P, Wang Y, Peng X, You G, Zhang W, Yan W, Bao Z, Wang Y, Qiu X, Jiang T (2013). Management and survival rates in patients with glioma in China (2004-2010): a retrospective study from a single-institution. J Neurooncol.

[5] Jiang T, Mao Y, Ma W, Mao Q, You Y, Yang X, Jiang C, Kang C, Li X, Chen L, Qiu X, Wang W, Li W, Yao Y, Li S, Li S (2016). CGCG clinical practice guidelines for the management of adult diffuse gliomas. Cancer Lett.

[6] Cheetham SW, Gruhl F, Mattick JS, Dinger ME (2013). Long noncoding RNAs and the genetics of cancer. Br J Cancer.

[7] Mercer TR, Dinger ME, Mattick JS (2009). Long non-coding RNAs: insights into functions. Nat Rev Genet.

[8] Fang Y, Fullwood MJ (2016). Roles, Functions, and Mechanisms of Long Non-coding RNAs in Cancer. Genomics Proteomics Bioinformatics.

[9] Bian EB, Li J, Xie YS, Zong G, Li J, Zhao B (2015). LncRNAs: new players in gliomas, with special emphasis on the interaction of lncRNAs With EZH2. J Cell Physiol.

[10] Sun Y, Wang Z, Zhou D (2013). Long non-coding RNAs as potential biomarkers and therapeutic targets for gliomas. Med Hypotheses.

[11] Zhang XQ, Leung GK (2014). Long non-coding RNAs in glioma: functional roles and clinical perspectives. Neurochem Int.

[12] Wang P, Ren Z, Sun P (2012). Overexpression of the long non-coding RNA MEG3 impairs in vitro glioma cell proliferation. J Cell Biochem.

[13] Wang Q, Zhang J, Liu Y, Zhang W, Zhou J, Duan R, Pu P, Kang C, Han L (2016). A novel cell cycle-associated lncRNA, HOXA11-AS, is transcribed from the 5-prime end of the HOXA transcript and is a biomarker of progression in glioma. Cancer Lett.

[14] Han Y, Wu Z, Wu T, Huang Y, Cheng Z, Li X, Sun T, Xie X, Zhou Y, Du Z (2016). Tumor-suppressive function of long noncoding RNA MALAT1 in glioma cells by downregulation of MMP2 and inactivation of ERK/MAPK signaling. Cell Death Dis.

[15] Zhang X, Sun S, Pu JK, Tsang AC, Lee D, Man VO, Lui WM, Wong ST, Leung GK (2012). Long non-coding RNA expression profiles predict clinical phenotypes in glioma. Neurobiol Dis.

[16] Heagerty PJ, Lumley T, Pepe MS (2000). Time-dependent ROC curves for censored survival data and a diagnostic marker. Biometrics.

[17] Zhang CB, Zhu P, Yang P, Cai JQ, Wang ZL, Li QB, Bao ZS, Zhang W, Jiang T (2015). Identification of high risk anaplastic gliomas by a diagnostic and prognostic signature derived from mRNA expression profiling. Oncotarget.

[18] Bao ZS, Li MY, Wang JY, Zhang CB, Wang HJ, Yan W, Liu YW, Zhang W, Chen L, Jiang T (2014). Prognostic value of a nine-gene signature in glioma patients based on mRNA expression profiling. CNS neuroscience & therapeutics.

[19] Cai J, Zhang W, Yang P, Wang Y, Li M, Zhang C, Wang Z, Hu H, Liu Y, Li Q, Wen J, Sun B, Wang X (2015). Identification of a 6-cytokine prognostic signature in patients with primary glioblastoma harboring M2 microglia/macrophage phenotype relevance. PloS one.

[20] Subramanian A, Tamayo P, Mootha VK, Mukherjee S, Ebert BL, Gillette MA, Paulovich A, Pomeroy SL, Golub TR, Lander ES, Mesirov JP (2005). Gene set enrichment analysis: a knowledge-based approach for interpreting genome-wide expression profiles. Proc Natl Acad Sci U S A.

[21] Wu Y, Liu H, Shi X, Yao Y, Yang W, Song Y (2015). The long non-coding RNA HNF1A-AS1 regulates proliferation and metastasis in lung adenocarcinoma. Oncotarget.

[22] Yang YC, Di C, Hu B, Zhou M, Liu Y, Song N, Li Y, Umetsu J, Lu ZJ (2015). CLIPdb: a CLIP-seq database for protein-RNA interactions. BMC Genomics.

[23] Li JH, Liu S, Zhou H, Qu LH, Yang JH (2014). starBase v2. 0: decoding miRNA-ceRNA, miRNA-ncRNA and protein-RNA interaction networks from large-scale CLIP-Seq data. Nucleic Acids Res.

[24] Yang JH, Li JH, Shao P, Zhou H, Chen YQ, Qu LH (2011). starBase: a database for exploring microRNA-mRNA interaction maps from Argonaute CLIP-Seq and Degradome-Seq data. Nucleic Acids Res.

[25] Figarella-Branger D, Colin C, Coulibaly B, Quilichini B, Maues De Paula A, Fernandez C, Bouvier C (2008). [Histological and molecular classification of gliomas]. Rev Neurol (Paris).

[26] Davis FG, Malmer BS, Aldape K, Barnholtz-Sloan JS, Bondy ML, Brannstrom T, Bruner JM, Burger PC, Collins VP, Inskip PD, Kruchko C, McCarthy BJ, McLendon RE, Sadetzki S, Tihan T, Wrensch MR (2008). Issues of diagnostic review in brain tumor studies: from the Brain Tumor Epidemiology Consortium. Cancer Epidemiol Biomarkers Prev.

[27] Josa-Prado F, Henley JM, Wilkinson KA (2015). SUMOylation of Argonaute-2 regulates RNA interference activity. Biochem Biophys Res Commun.

[28] Kim JK, Jin X, Ham SW, Lee SY, Seo S, Kim SC, Kim SH, Kim H (2015). IRF7 promotes glioma cell invasion by inhibiting AGO2 expression. Tumour Biol.

[29] Guo J, Lv J, Liu M, Tang H (2015). miR-346 Up-regulates Argonaute 2 (AGO2) Protein Expression to Augment the Activity of Other MicroRNAs (miRNAs) and Contributes to Cervical Cancer Cell Malignancy. J Biol Chem.

[30] Bian XJ, Zhang GM, Gu CY, Cai Y, Wang CF, Shen YJ, Zhu Y, Zhang HL, Dai B, Ye DW (2014). Down-regulation of Dicer and Ago2 is associated with cell proliferation and apoptosis in prostate cancer. Tumour Biol.

[31] Kim S, Yamamoto J, Chen Y, Aida M, Wada T, Handa H, Yamaguchi Y (2010). Evidence that cleavage factor Im is a heterotetrameric protein complex controlling alternative polyadenylation. Genes Cells.

[32] Verghese ET, Drury R, Green CA, Holliday DL, Lu X, Nash C, Speirs V, Thorne JL, Thygesen HH, Zougman A, Hull MA, Hanby AM, Hughes TA (2013). MiR-26b is down-regulated in carcinoma-associated fibroblasts from ER-positive breast cancers leading to enhanced cell migration and invasion. J Pathol.

[33] Huang YH, Peng W, Furuuchi N, Gerhart JV, Rhodes K, Mukherjee N, Jimbo M, Gonye GE, Brody JR, Getts RC, Sawicki JA (2016). Delivery of therapeutics targeting the mRNA-binding protein HuR using 3DNA nanocarriers suppresses ovarian tumor growth. Cancer Res.

[34] Melling N, Taskin B, Hube-Magg C, Kluth M, Minner S, Koop C, Grob T, Graefen M, Heinzer H, Tsourlakis MC, Izbicki J, Wittmer C, Huland H (2016). Cytoplasmic accumulation of ELAVL1 is an independent predictor of biochemical recurrence associated with genomic instability in prostate cancer. Prostate.

[35] Brooke GN, Culley RL, Dart DA, Mann DJ, Gaughan L, McCracken SR, Robson CN, Spencer-Dene B, Gamble SC, Powell SM, Wait R, Waxman J, Walker MM, Bevan CL (2011). FUS/TLS is a novel mediator of androgen-dependent cell-cycle progression and prostate cancer growth. Cancer Res.

[36] Ward CL, Boggio KJ, Johnson BN, Boyd JB, Douthwright S, Shaffer SA, Landers JE, Glicksman MA, Bosco DA (2014). A loss of FUS/TLS function leads to impaired cellular proliferation. Cell Death Dis.

[37] Wang W, Zhang L, Wang Z, Yang F, Wang H, Liang T, Wu F, Lan Q, Wang J, Zhao J (2016). A three-gene signature for prognosis in patients with MGMT promoter-methylated glioblastoma. Oncotarget.

