# Supplementary material for: LncRNA profile study reveals four-lncRNA signature associated with the prognosis of patients with anaplastic gliomas
Source: Oncotarget. 2016 Oct 13;7(47):77225–36. doi: 10.18632/oncotarget.12624 (PMC5363582; doi:10.18632/oncotarget.12624)
Supplement: Supplementary file 1 [file oncotarget-07-77225-s001.pdf]

LncRNA profile study reveals four-lncRNA signature associated with the prognosis of patients with anaplastic gliomas

SUPPLEMENTARY TABLES

Supplementary Table S1: Clinicopathologic factors associated with OS in the Cox regression analysis for patients from the CGGA and REMBRANDT dataset

Variable	Univariate Cox		Multivariate Cox	
	p-value	HR	p-value	HR
Age	0.243	1.020	0.198	0.939
Gender	0.606	1.241		
KPS	0.457	0.985	0.303	1.034
IDH1	0.317	0.655		
Chemotherapy	0.150	0.499		
Risk Score	0	1.578	0.030	1.838

Gender, male 1, female 2; IDH1 mutation status, mutated 1, wild-type 0; Chemotherapy, Yes 1, No 0.

REMBRANDT microarray dataset

Variable	Univariate Cox		Multivariate Cox	
	p-value	HR	p-value	HR
Gender	0.813	1.080	0.911	1.037
Risk Score	0	2.039	0	2.503

Gender, male 1, female 2.

Supplementary Table S2: Four-lncRNA primers list and AGAP2-AS1 siRNA oligos sequences.

See Supplementary File 1
