# Supplementary material for: LncRNA profile study reveals four-lncRNA signature associated with the prognosis of patients with anaplastic gliomas
Source: Oncotarget. 2016 Oct 13;7(47):77225–36. doi: 10.18632/oncotarget.12624 (PMC5363582; doi:10.18632/oncotarget.12624)
Supplement: Supplementary file 2 [file oncotarget-07-77225-s002.docx]

**Primer list:**

AGAP2-AS1-F: 5'-GGAGAGGGAGCTCACGTACC-3'

AGAP2-AS1-R: 5'-TGGCTACCGTTCTCCTGCTT-3'

TPT1-AS1-F: 5'-CACTCCCAGATCTTCACTTCAGG-3'

TPT1-AS1-R: 5'-AATTGGAGGCCAGTGCTCTG-3'

LINC01198-F: 5'-TGACAGGATAACTTCAGGAAGTGG-3'

LINC01198-R: 5'-TGCTGGACGATAACAATGGC-3'

MIR155HG-F: 5'-TGGCACTGCAGATAACTTGTCTG-3'

MIR155HG-R: 5'-ATGTAGGAGTCAGTTGGAGGCAA-3'

GAPDH-F: 5'-AGGGCTGCTTTTAACTCTGGT-3'

GAPDH-R: 5'-CCCCACTTGATTTTGGAGGGA-3'

**SiRNA list:**

AGAP2-AS1-Homo-siRNA1

5'-CUGCAAGACCAGGGAUCAATT-3'

5'-UUGAUCCCUGGUCUUGCAGTT-3'

AGAP2-AS1-Homo-siRNA2

5'-CAAACUCUUACCUUGACCUTT-3'

5'-AGGUCAAGGUAAGAGUUUGTT-3'

AGAP2-AS1-Homo-siRNA3

5'-CAUUAAGGGACAGAGUUCATT-3'

5'-UGAACUCUGUCCCUUAAUGTT-3'

Negative control

5'-UUCUCCGAACGUGUCACGUTT-3'

5'-ACGUGACACGUUCGGAGAATT-3'
